# Appendix

**DOI:** 10.1007/s13280-014-0567-y

**Published:** 2014-11-15

**Figure:**
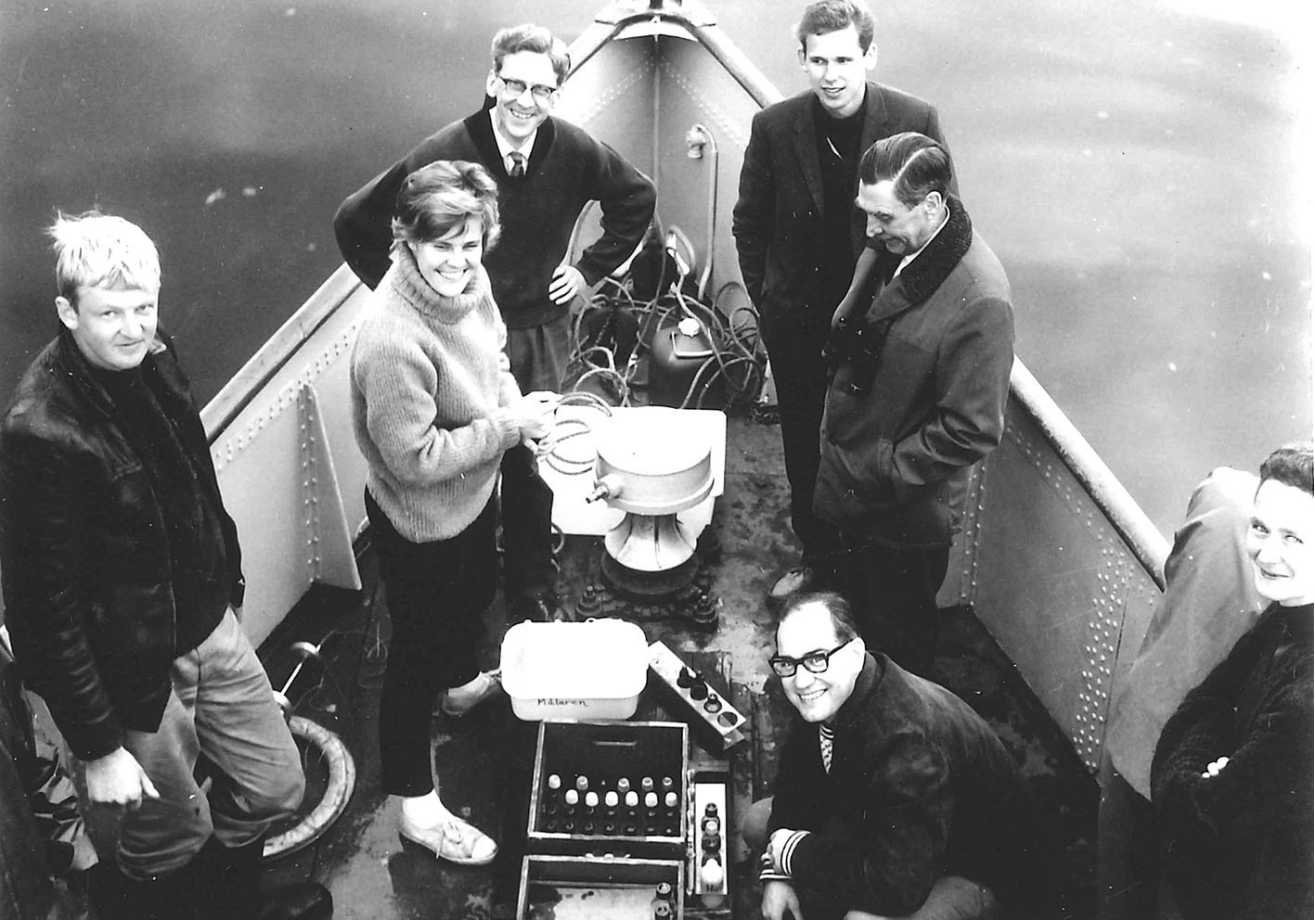
Torsten Ahl (squatting, *bottom right*) and Torbjörn Willén (*upper left*), two pioneers of the Swedish national freshwater monitoring on the investigation of Swedish great lakes. Unknown photographer

**Figure:**
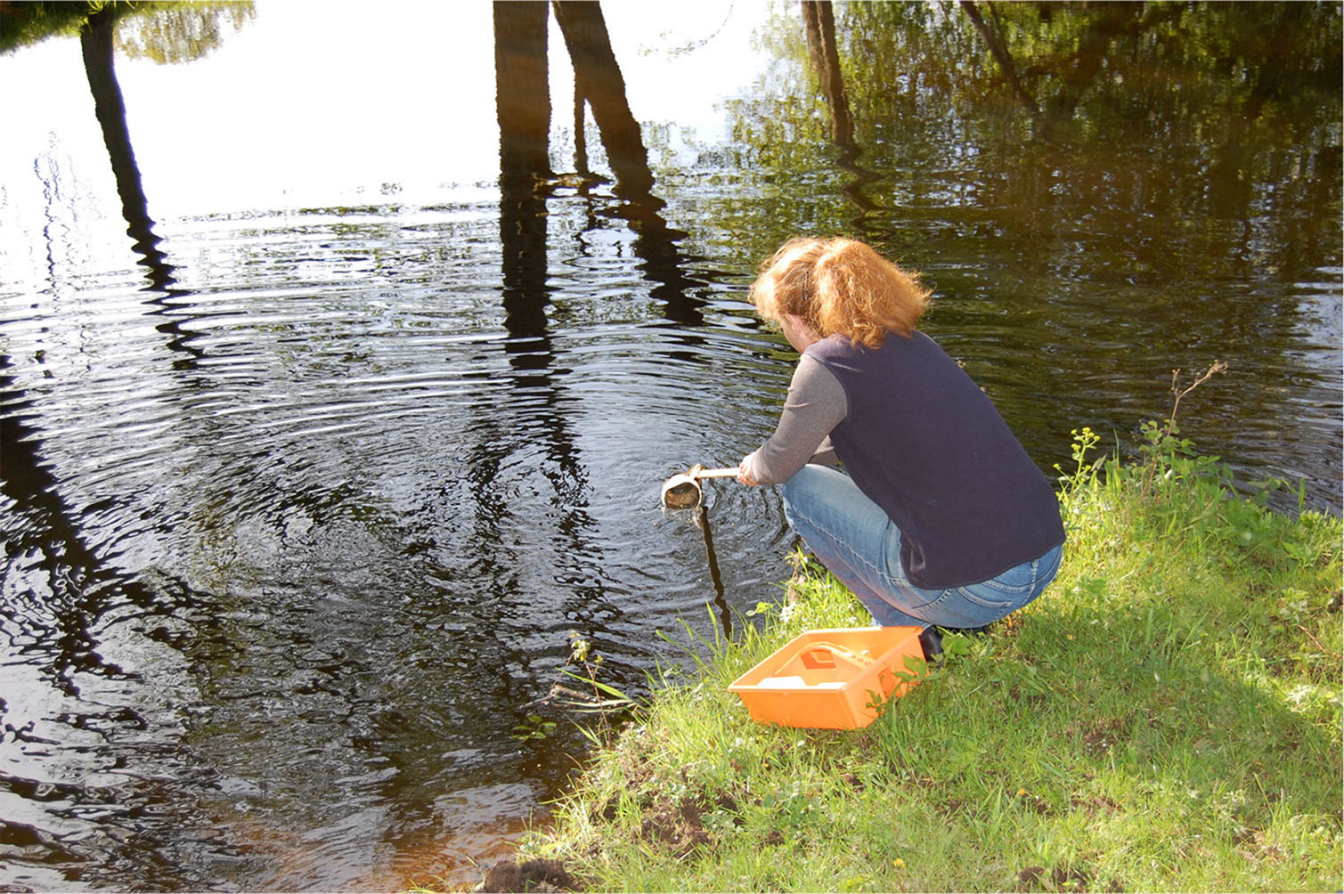
Christel Lindholm is one of the local inhabitants who each month takes a water sample and sends to the lab for analyses of water chemistry. Photo by Leif Lindholm

**Figure:**
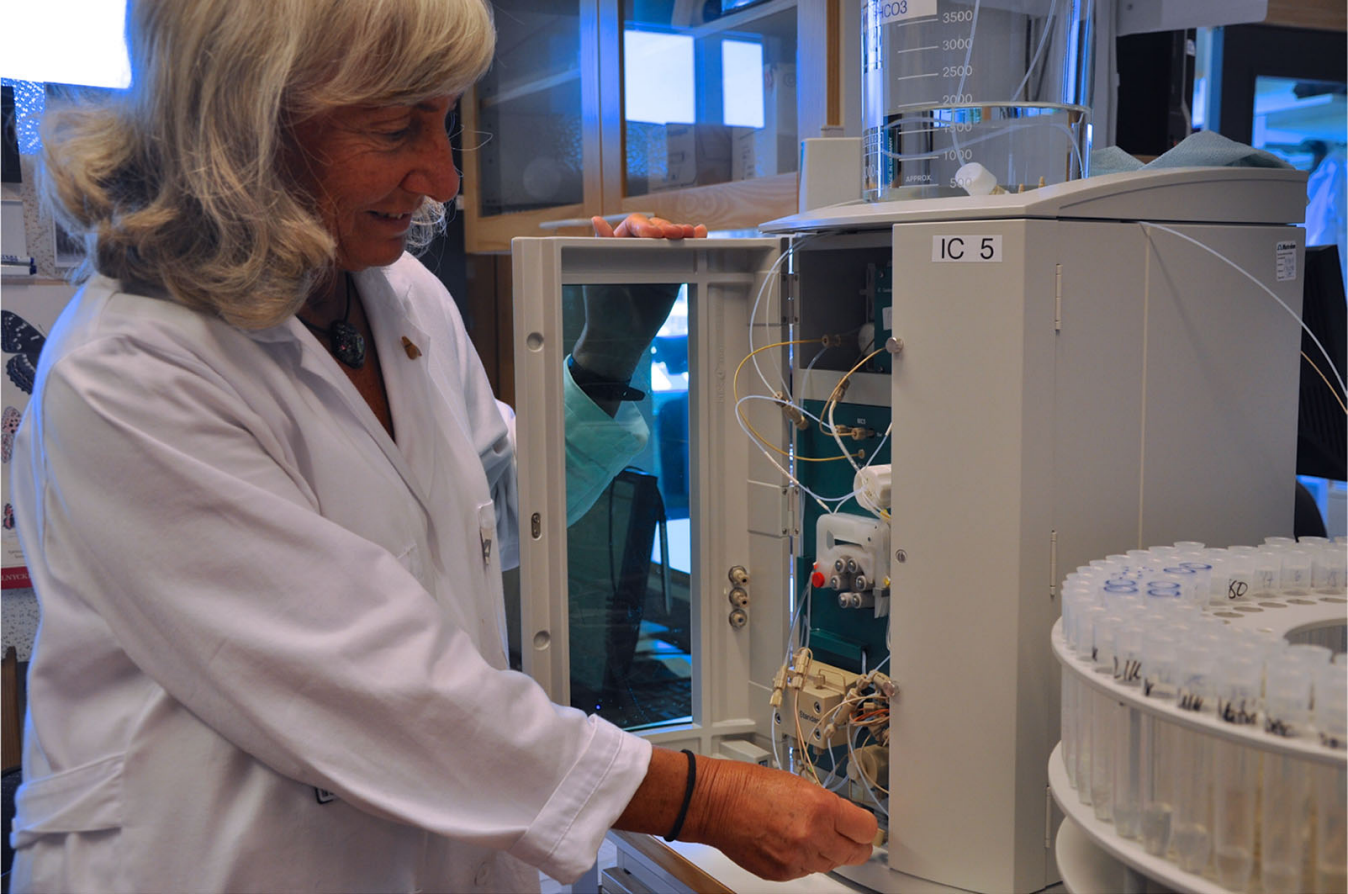
Gun Hölling who has worked with environmental monitoring since 1971 with the ion chromatograph, which replaced the old Macereth method for sulfate in 1984. Photo by Jens Fölster

**Figure:**
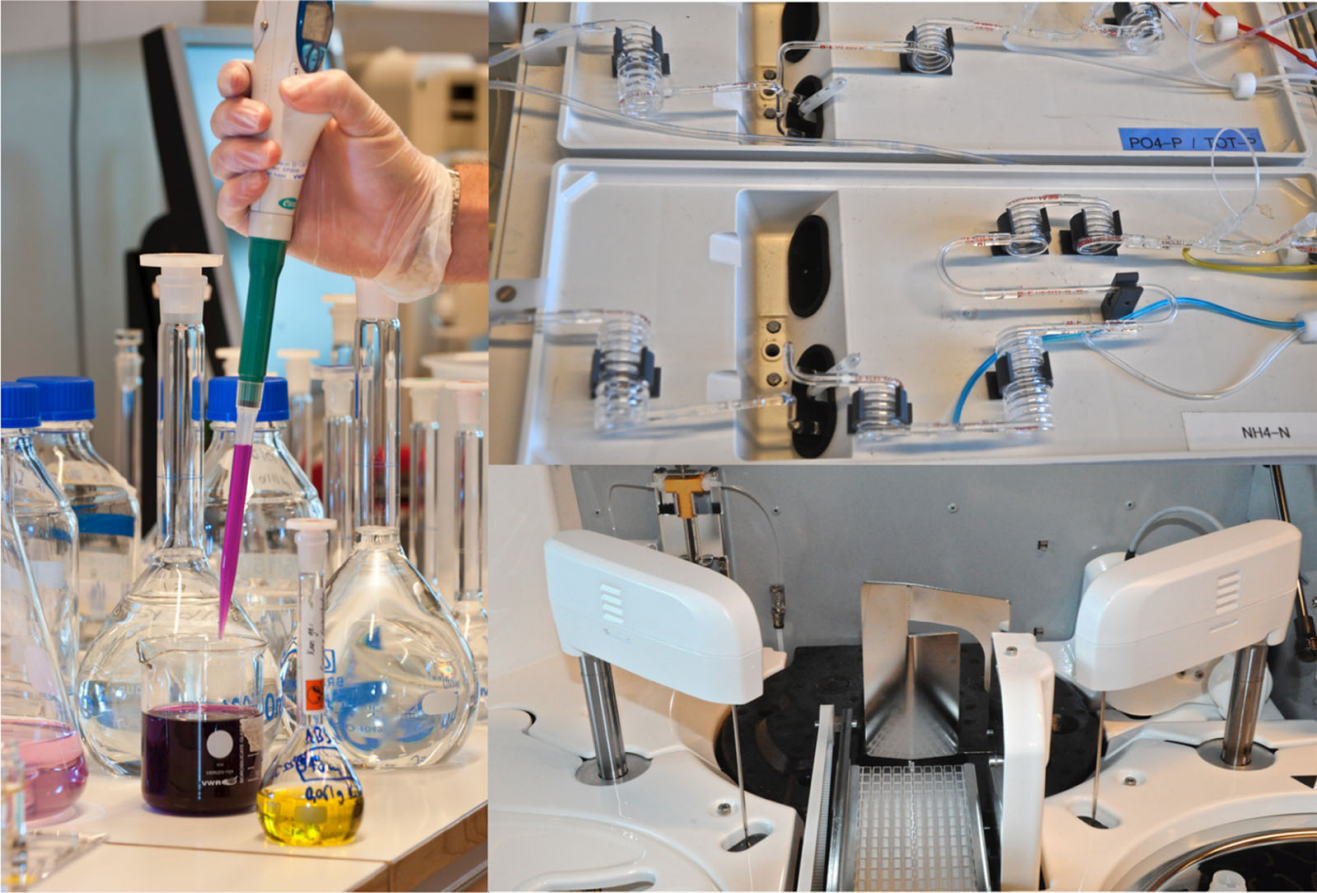
Three generations of analytical techniques: Hand-made permanganate consumption made to ensure the consistency of the long-term time series (*left*), auto-analyser (*upper right*), and a high-throughput discrete analyser (*lower right*). Photo by Jenny Svennås-Gullner (*left*) and Jens Fölster (*right*)

**Figure:**
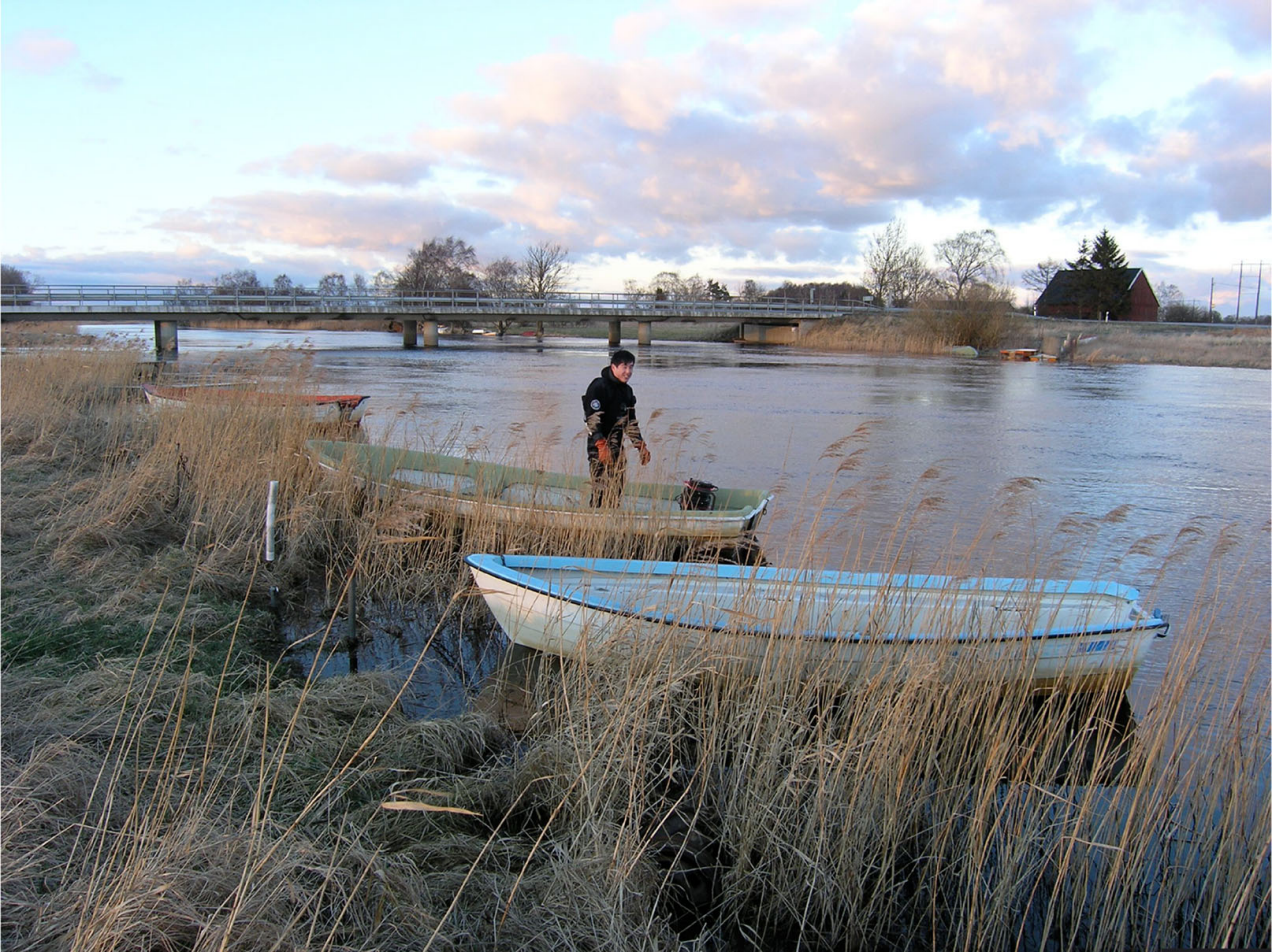
Jonas Pålsson from the consulting company *Al*-*control* taking a sample in Helgeån. Photo by Lars Göran Karlsson

**Figure:**
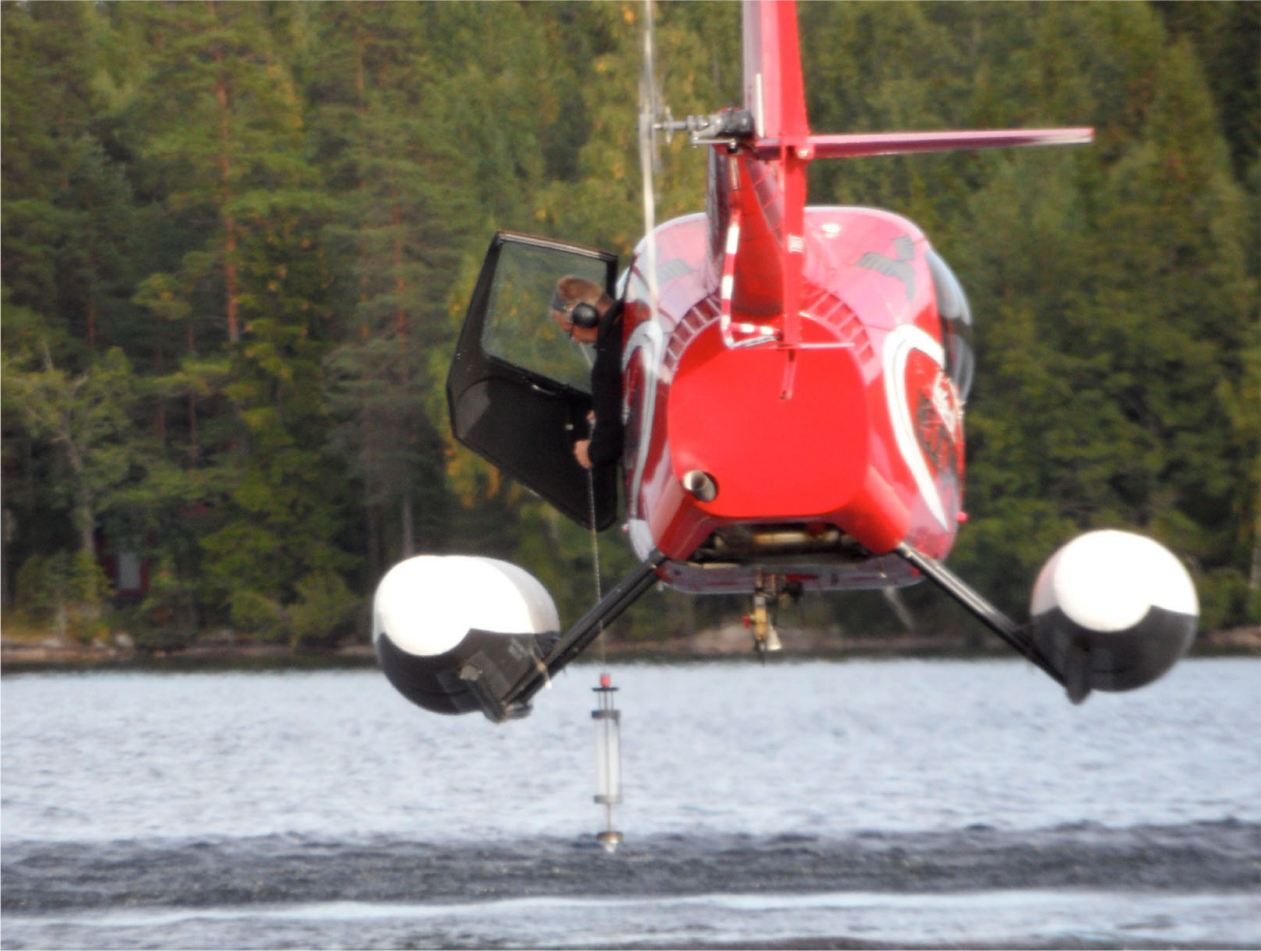
Helicopter sampling during the lake survey. Photo by Jens Fölster

**Figure:**
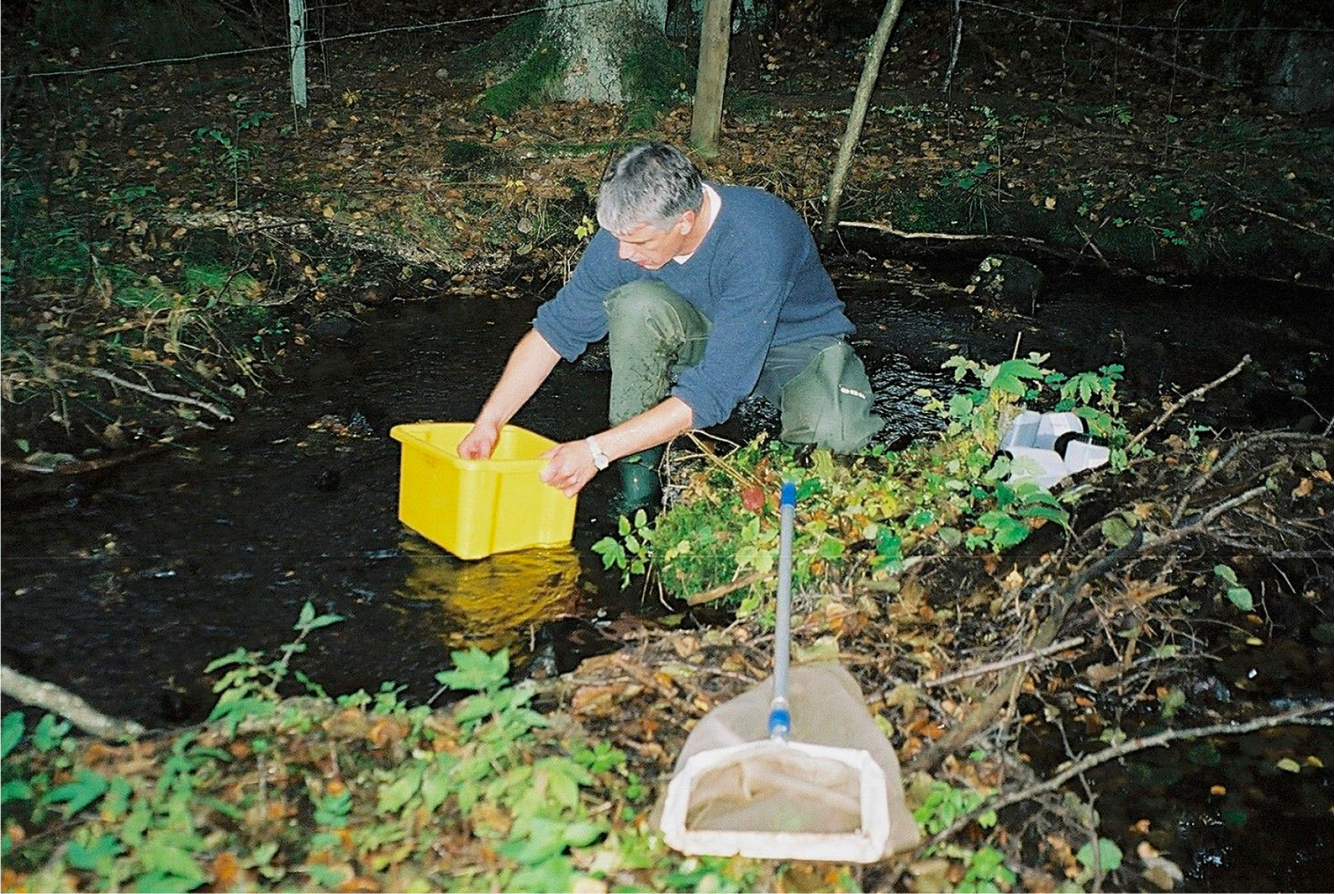
Willem Goedkoop taking a kick sample for benthic invertebrates. Photo by Stefan Löfgren

